# Supplementary figures and images for: Extracellular vesicles from mature dendritic cells (DC) differentiate monocytes into immature DC
Source: Life Sci Alliance. 2018 Dec 3;1(6):e201800093. doi: 10.26508/lsa.201800093 (PMC6277684; doi:10.26508/lsa.201800093)

Figure 3D

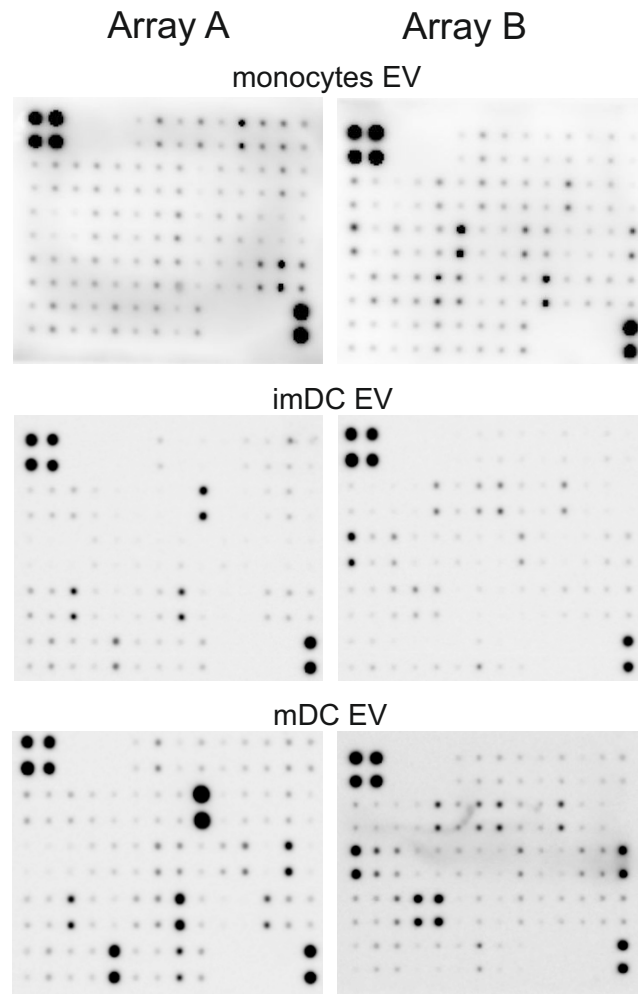

Figure S4

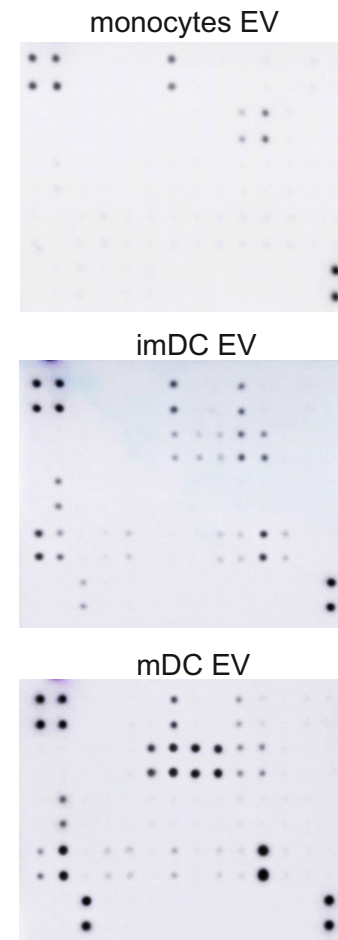

Supplement: Supplementary file 1 [file LSA-2018-00093_SdataF1.pdf]
