# Supplementary material for: Extracellular vesicles from mature dendritic cells (DC) differentiate monocytes into immature DC
Source: Life Sci Alliance. 2018 Dec 3;1(6):e201800093. doi: 10.26508/lsa.201800093 (PMC6277684; doi:10.26508/lsa.201800093)
Supplement: Supplementary file 2 [file LSA-2018-00093_TableS1.pdf]

		after 24 h iDC	after 24 h mDC
cells in EV-area	[µm²]	7320	13455
total:	[n]	399	609
$CD45^{+}$:	[n]	296	607
	[%]	74.19	99.7
$CD11^{+}$/$Ly6C^{+}$/$Ly6^{-}$:	[n]	12	255
	[%]	3.1	42.1
